# Digital health interventions for suicide prevention among LGBTQ: A narrative review

**DOI:** 10.3126/hprospect.v23i1.62795

**Published:** 2024-03-11

**Authors:** Kiran Paudel, Kamal Gautam, Prashamsa Bhandari, Sangam Shah, Jeffrey A Wickersham, Bibhav Acharya, Sabitri Sapkota, Samir Kumar Adhikari, Phanindra Prasad Baral, Archana Shrestha, Roman Shrestha

**Affiliations:** 1 Department of Allied Health Sciences, University of Connecticut, Storrs, CT 06269, USA; 2 Nepal Health Frontiers, Tokha-5, Kathmandu, Nepal; 3 Institute of Medicine, Tribhuwan University, Kathmandu, Nepal; 4 Department of Internal Medicine, Section of Infectious Diseases, Yale School of Medicine, New Haven, CT, USA; 5 Department of Psychiatry and Behavioral Sciences, UCSF Weill Institute for Neurosciences, School of Medicine, 675 18th Street, San Francisco, CA, 94107, USA; 6 Possible, a non-profit organization, Bhim Plaza, Kathmandu, Nepal; 7 Ministry of Health and Population, Ram Shah Path, Kathmandu, Nepal; 8 Non-communicable Disease and Mental Health Section, EDCD, DOHS, MOHP, Nepal; 9 Department of Public Health, Kathmandu University School of Medical Sciences, Dhulikhel, Nepal

**Keywords:** Digital interventions, LGBTQ, suicide interventions, narrative review

## Abstract

**Background:**

Suicidal thoughts and behaviors (STBs) are prevalent within the Lesbian, Gay, Bisexual, Transgender, and Queers (LGBTQ) community, often exacerbated by challenges in accessing care and the perceived stigma and discrimination tied to disclosing one’s identity. Digital health interventions that offer psychosocial self-help present a promising platform to reach individuals at risk of STBs, especially those who may not engage with conventional health services. This review aimed to assess the role of digital-based intervention in reducing STBs among LGBTQ individuals.

**Methods:**

We conducted a systematic literature search from three databases, PsycINFO, PubMed, and CINHAL, from 1st Jan 1990 to 31st December 2023. The review encompassed studies investigating the feasibility, acceptability, and impact of digital interventions on STBs, employing randomized control trials (RCTs), pseudo-RCTs, observational pre-posttest designs, and qualitative studies. Potential bias was evaluated using the McGill Mixed Methods Appraisal Tool (MMAT).

**Results:**

Five non-overlapping studies were included, reporting data from 777 participants. The studies featured diverse types of digital interventions, including videos, online writing, and mobile applications. The studies included three RCTs, and two qualitative studies. Across most of these studies, notable enhancements or reductions in the proportion of participants reporting STBs were observed post-intervention, alongside improvements in help-seeking intentions. The findings underscored that the applications used in the studies were engaging, acceptable, and deemed feasible in effectively addressing suicide prevention among the LGBTQ community.

**Conclusion:**

Overall, digital interventions were found to be feasible and acceptable in suicide prevention among LGBTQ communities, demonstrating preliminary efficacy in increasing help-seeking behavior when experiencing suicidal thoughts and in reducing STBs. Therefore, advocating for widespread promotion and dissemination of digital health interventions is crucial, particularly in low- and middle-income countries (LMICs) with limited access to health services and heightened barriers to obtaining such services. Further research using fully powered RCT is imperative to assess the efficacy of these interventions.

## Tweetable abstract

Digital interventions were found to be feasible and acceptable in suicide prevention among LGBTQ.

## Introduction

Suicide is a serious global public health issue and is among the leading causes of death worldwide [1]. As research on health outcomes within the sexual and gender minorities population becomes increasingly available, it is evident that this group is at elevated risk of suicide as compared to their heterosexual and cisgender counterparts [2, 3]. Health disparities between these marginalized groups and their peers can largely be attributed to specific minority stressors such as stigma, discrimination, victimization, internalized homophobia [4], and pressure to conform to heteronormativity. Additionally, these disparities are influenced by the typical general stressors (ecological, interpersonal, and behavioral factors) [3]. These multi-level factors contribute to diminished psychological well-being, thereby elevating the risk of adverse health outcomes such as suicide and suicidal behaviors.

Various interventions, such as brief contact intervention, internet-based interventions, group therapies, pharmacotherapy-related interventions (e.g., lithium treatment or short-term intravenous ketamine), counseling, mental health promotion, stress management, coping support, and cognitive behavioral therapy, are found to be competent and effective in the prevention of suicidal behaviors [5, 6]. There is a significant lack of interventions specifically tailored to the distinctive needs of the Lesbian, Gay, Bisexual, Transgender, and Queers (LGBTQ) community [7]. This lack of targeted interventions in suicide prevention among LGBTQ individuals poses a significant threat to their interpersonal and socio-economic lives. Recognizing the urgent need for tailored interventions, it is imperative to explore novel approaches that can address the unique challenges faced by the LGBTQ population.

Digital interventions, a prominent facet of the contemporary healthcare system, have recently gained attention for their potential use in healthcare service delivery. They focus on enhancing health outcomes through technological means, including the internet, virtual care, artificial intelligence, and intelligent wearables [8]. These interventions, delivered through web-based programs or mobile applications, have found applications in suicide prevention and management [9], offering multiple methods to provide instructions and skills training, including email, text messages, video clips, and online modules [9]. Their potential to provide therapeutic support at users’ discretion, anonymously, and cost-effectively makes digital interventions a scalable solution [9], particularly addressing resource limitations in low- and middle-income countries [10].

In the context of LGBTQ individuals who continue to face societal resistance [4], digital interventions provide a discreet and private space for engagement while maintaining confidentiality and anonymity [11, 12]. These interventions can be personalized by leveraging algorithms to cater to each individual’s unique experiences [13]. The benefits of digital interventions in actively engaging participants and maintaining a higher level of adherence [14] make them a crucial component in mitigating suicidal thoughts and behaviors (STBs) among the LGBTQ population.

Despite the potential benefits of digital interventions, there is a gap in the literature regarding their effectiveness for the LGBTQ population, emphasizing the need for a comprehensive review. Through a mixed methods approach, combining both quantitative and qualitative evidence, this narrative review aims to provide an understanding of the effectiveness of digital interventions, focusing on their potential as an essential tool in suicide prevention efforts within the LGBTQ population.

This narrative review aims to identify, systematically review, and synthesize published articles describing interventions to prevent suicidal behavior and reduce suicidal ideation in LGBTQ. Feasibility, acceptability, and usability of digital interventions were examined for improving STBs within a randomized control trial, observational pre-posttest design, and qualitative studies.

## Material and methods

### The PICO framework

The review adhered to the WHO 2nd edition of the guideline development process, involving formulating critical questions using the PICO format (population, intervention, comparator, and outcomes). Subsequently, a scoping review was conducted to identify any existing systematic reviews capable of addressing the key questions and establishing grounds of evidence [14]. Based on the scoping review findings, the decision-making process involves whether a relevant review already exists, whether an existing review requires updating, or whether an entirely new review needs to be initiated.

During our research, we found that there is no existing review paper done on digital health interventions for improving STBs among LGBTQ. The guideline development process includes defining the PICO framework, leading to the meaning of the critical question. Table 1 showcases the components on which the following fundamental question was formulated:

### Are digital health interventions helpful for suicide prevention among LGBTQ people?

#### Search strategy and selection criteria

This review adhered to the Preferred Reporting Items for Systematic Reviews and Meta-Analyses (PRISMA) guidelines. Extensive search strategies were employed utilizing specific keywords across multiple databases, including Web Science, PubMed, and Scopus. The focus of this research was on suicide, the LGBTQ community, and digital health interventions. During the database search, temporal limitations were not applied; data were searched from 1^st^ Jan 1990 to 31^st^ Dec 2023. The list of keywords and full search strategy are detailed in Table 2.

##### Inclusion Criteria:

Quantitative, qualitative, and mixed-methods studies published in peer-reviewed journals, and in English language. Articles reporting at least one group of sexual or gender minorities as the targeted population of interventions. The studies focused exclusively on STB and evaluated interventions delivered through digital platforms like websites or apps.

##### Exclusion Criteria:

Letters to the editor, review articles, conference abstracts, dissertations or theses, and studies published in languages other than English were excluded. Studies focusing on non-digital mediums were also excluded from the review.

#### Data extraction

Three authors (KP, KG, and PB) extracted data from the included studies and one author (SS) verified the extracted data. Disagreements were resolved by discussion among the authors.

#### Quality assessment

McGill Mixed Methods Appraisal Tool (MMAT) was used to assess the risk of bias in mixed method research studies [15]. The MMAT was designed to help researchers critically evaluate quantitative and qualitative studies within the context of mixed methods systematic reviews. This tool has been validated as reliable and effective for this purpose. MMAT has five criteria for five study designs (randomized control trial, quantitative randomized control trial, quantitative nonrandomized trial, qualitative and quantitative randomized trials, quantitative descriptive, and mixed methods) to evaluate studies. After this, the MMAT assigns an overall quality score to each study by dividing the number of positive criteria met by 5. A quality assessment using a five-point checklist found diverse scores among included studies, ranging from low (1 out of 5 criteria met) to high (all 5 criteria met). KP and KG independently conducted the assessment, and discrepancies were resolved through discussion. No studies were excluded based on quality. Out of five identified studies, 40% (2/5) were rated as poor quality while 60% (3/5) were rated as high quality.

#### Data synthesis

Characteristics of each study were synthesized in the summary table. While summarizing data, descriptive statistics (frequencies and percentages for dichotomous variables; mean for continuous variables) were calculated, as shown in Table 3.

## Results

### Literature search

**Figure 1:**
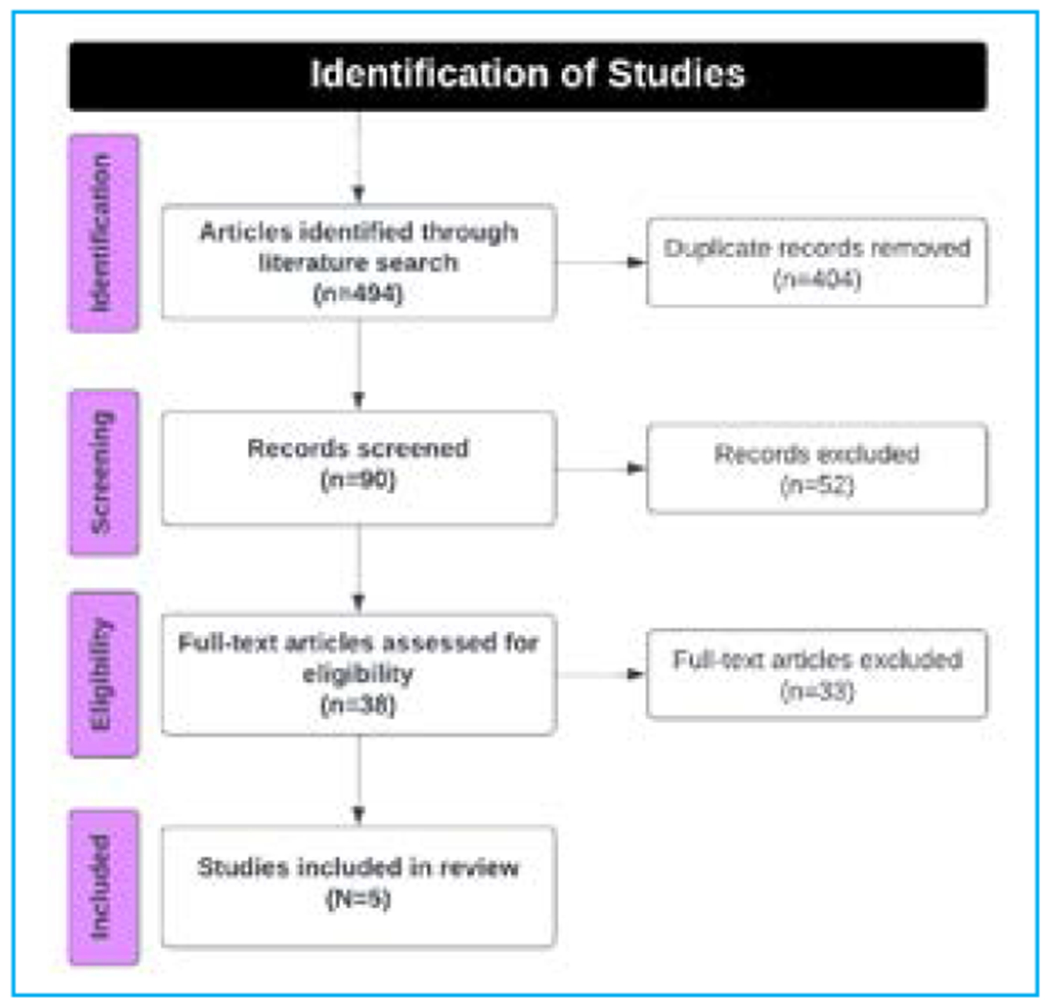
Preferred reporting items for narrative reviews for article identification and selection (PRISMA Guideline)

The literature search yielded 494 articles. After removing duplicates and excluding studies based on abstract followed by full text as eligible for inclusion, as shown in Figure 1.

Many of these studies were conducted in the United States, whereas one was conducted in China [16–19]. One study was conducted in multiple countries (i.e., Austria and Germany) [20]. This study was promoted through LGBTQ organizations in Austria and Germany using LGBTQ Facebook groups, Reddit, or Tumblr pages targeting LGBTQ. The online part of the study was conducted in German-speaking settings (i.e., Austria and Germany), while the on-site part was conducted in Vienna, Austria.

Four of the studies included individuals of had a mean age of the participants less than 24 years; however, one was 33.4 years old with a standard deviation of 9.5 [16, 17, 19, 20]. One of the studies was carried out among school-going students by involving parents whose mean age of the school-going participants was 15.7±1.3 years old (13-18), and the mean age of the parents was 48 years old [18]. Regarding study design, three articles were randomized controlled trials (RCT), and two were qualitative studies.

### Characteristics of evidence-based digital health interventions.

Out of five studies on digital health interventions, two of them used online videos as the intervention for suicide prevention [19, 20]. Han, M., et.al used online animated psychoeducational videos facilitated by led group discussion, and electronic help seeking brochures whereas Kirchner, S., et.al. features a young cisgender man and women to strengthen sexual identity and prevent science [19, 20]. Two of the other studies developed mobile phone applications, and one used brief online writing intervention and self-affirmation [16–18]. In study done by Pachankis, JE., et.al. for brief online writing interventions, one writes about a stressful or traumatic event for about 20 minutes per day for several days.

And for self-affirmation three brief scenarios reflecting common instances of severe sexual minority stress: family rejection, religious community rejection, and school-based victimization. Participants in the self-affirmation group were tasked with a daily 20-minute writing exercise for three consecutive days [16].

### Acceptability and feasibility of current digital health interventions

Out of five studies, two qualitative studies examined the acceptability and feasibility of the mobile application [17, 18]. In one study done by Biernesser, C., et.al. digital platforms were prepared by active participation with the participants. Both studies reported that applications were engaging, acceptable, and feasible in suicide prevention. One of the studies was done among youth and parents, and all youth reported that digital intervention would be easy to use without parents.

A study involving actively engaged LGBTQ youth found that all participants perceived the Flourish app as easy to use independently, without parental assistance. The System Usability Scale (SUS) mean score was 91 (on a scale of 0-100), indicating a high level of usability [18]. The participants expressed interest in using the app due to its positive, caring, empathetic, authentic, and relatable tone, finding it helpful for coping skills. Notably, all participants believed the app would be easy to learn, with a couple suggesting the inclusion of instructions for improved understanding among adolescents. While many users did not track their mood daily (averaging four days per week), they logged in approximately four times a week, spending an average of 5 minutes per session. Overall, this review suggests that the mobile app is an engaging and acceptable mental health intervention, providing initial support for its acceptability and usability.

### Suicidal ideation

Three of the studies included results for self-reported suicidal ideation [16, 19, 20]. In most of the studies, as post-intervention, there was an improvement or reduction in the proportion of participants self-reporting suicidal ideation after the interventions. However, in the study by Kirchner, S., et.al. there was no effect of exposure to the videos on the total intervention group regarding suicidal ideation improvement [20]. Still, there was an improvement in help-seeking intentions compared to controls. In a study done by Han, M., et.al., there was an improvement in help-seeking intention of suicidal ideation [19]. Help seeking intention during the mood swings can help in the suicide prevention, if they people have negative feeling at that time [17]. A qualitative study by Dubov, A A., et.al. reported that:

*“Mood logging made me more aware of my feelings, how my mood changes over time, and how to deal with mood changes. Even when I feel down, I can remind myself that these feelings are normal and will likely pass.”* [17]

## Discussion

The findings of this narrative review showed a global landscape of digital health interventions for the reducing suicidal ideation and preventing suicide among LGBTQ persons using different research designs across various age groups. Digital health interventions like mobile applications, online videos, and brief online self-affirmation writing were used for suicide prevention among LGBTQ. A noteworthy observation is that most articles scored low on quality according to the MMAT criteria [15].

Studies of digital health interventions among LGBTQ have been primarily conducted in developed countries, especially the USA [16–20]. These trends denote that digital health interventions are widely implemented in developed countries, which are resource-rich settings. This can be potentially attributed to general perceptions that low-and-middle-income countries (LMIC) face difficulties adopting technology-based interventions due to less digital-friendly policies, limited internet access, data insecurity challenges, and privacy threats [21–23]. However, recent progress in digital accessibility in LMICs offers promising opportunities to bridge the technological disparities and promote global digital health equity [21, 22, 24]. Institutional support is required in many LMICs to overcome these challenges and develop and consolidate digital health strategies [25, 26]. The WHO guidelines with ten evidence-based recommendations for strengthening health systems could greatly help LMICs to implement digital health interventions [25].

Pachankis, JE. et.al. found that participants who received the intervention showed significant improvement in help-seeking intentions for suicidal ideation compared to the control group at the one-month follow-up [16]. These improvements were higher than those observed in similar studies involving populations other than LGBTQ [27–29], indicating greater efficacy of digital health interventions in the LGBTQ community. Another similar study in LGBTQ observed improvement in help-seeking intentions; however, there was no significant change in suicidal ideation [20]. This lack of change in suicidal ideation may be attributed to the anticipation that behavioral shifts would manifest in the long term rather than within months [30]. The emphasis on coping with adversity in the video-based intervention content without an explicit focus on suicide prevention could have contributed to the absence of change in suicidal ideation.

To explore the long-term impact of digital interventions, it is strongly recommended that future studies be conducted over an extended period to observe potential changes in suicidal ideation [19, 20]. Additionally, there is no clear indication that interventions based on a particular modality (e.g., videos, mobile apps) are more effective. Consequently, comprehensive research remains essential for a thorough understanding of the efficacy of various intervention modalities within the study population.

Study done by Biernesser, C., et.al. also found that the interventions identified in this review involved expert stakeholders and community members in designing or delivering the digital interventions [18]. This ensures that the interventions are culturally competent, relevant, and sensitive to the unique challenges faced by the community. It also fosters a sense of ownership and probability of sustainable outcomes of the interventions. Likewise, the interventions were tailored around the needs and minority stressors of the LGBTQ people. Some studies included the ‘coming out’ aspect, while others focused on addressing the needs of participants in rural areas with additional needs due to fewer coping resources. This might be one of the reasons for the demonstrated efficacy of the digital health interventions included in this study.

Anonymity and privacy warranted by digital health interventions enable people to seek help without judgment or fear of facing stigma or disclosure of identity, making these interventions more impactful among those populations than the general population [28, 31]. These interventions can also become safe platforms for community building and peer support and provide spaces where people do not have to face institutional barriers, which is more aggravated by the minority stress in the LGBTQ population. Therefore, technology-enhanced suicide prevention interventions warrant additional exploration as it is a relatively new area for suicide prevention. Despite exhaustive searches, limited evidence is available. No studies compared a technology-based enhanced treatment group to face-to-face treatment. However, it is essential to explore whether digital interventions complement traditional face-to-face approaches or can be beneficial as a stand-alone intervention. Study findings suggested that digital health is an integral part of health priorities, and people can benefit in an ethical, safe, secure, reliable, equitable, and sustainable way.

Self-guided digital health interventions like social media-based, telehealth-based, text messages, and online video-based were found to be successfully supporting LGBTQ individuals [16–19]. Our findings showed digital health interventions were accepted and feasible to carry out among LGBTQ for suicide prevention. Prior research shows that the perceived and actual functionality of digital interventions can impact the acceptability of technological interventions [28]. Therefore, additional exploration is needed to explore LGBTQ’s unique preferences and attitudes toward digital interventions. Digital interventions tailored to LGBTQ and developed with their active participation in connecting with community-based interventions would be a significant and effective intervention for suicide interventions. Traditional forms of treatment with mental health professionals cannot meet the needs of the individuals [32]. In addition, digital health intervention can play a pivotal role in overcoming immense structural barriers to treatment, including stigma, financial cost, and attitudinal barriers [33]. Advanced technology that is user-friendly, accessible, and acceptable offers the opportunity to increase access to suicide prevention through novel digital formats and delivery.

This review highlights the need for research, development, and implementation strategies aligned with the unique needs of the LGBTQ population, ensuring interventions are ethical, safe, secure, reliable, equitable, and sustainable. Additionally, an emphasis on continuous evaluation of these digital interventions is needed for continuous improvement and adaptation to the community’s dynamic needs.

### Practice implications

As a part of suicide prevention efforts, we can and should promote digital interventions such as app stores that directly target suicidality. Even though the impact of these interventions may be small individually, the overall population impact could be significant if uptake is widespread. Various interventions, including dialectic behavior therapy, cognitive behavior therapy for insomnia, and therapeutic evaluative conditioning, are likely more effective than general cognitive behavioral therapy alone. This highlights the need for suicide prevention content in novel ways within apps and online programs for effective treatment. Just-in-time Intervention (JITAIs), which uses smartphones and wearable, offers a promising pathway towards effective suicide prevention among the general population, which could be more effective and needed more in LGBTQ [33]. Digital health-based interventions can be developed with the principles of accessibility, scalability, replicability, transparency, privacy, security, and confidentiality.

There are subpopulation-specific needs owing to the unique societal challenges of being a sexual minority versus a gender minority. These can be compounded by other intersections such as culture, ethnicity, and age. Therefore, while developing the app or carrying out digital health interventions, essential features and tailoring of the content of other subpopulations may be required, which is underreported in the studies and this narrative review. Future research must depict the needs and essential features of different sub-populations within the LGBTQ community.

### Limitations

Our study has several limitations. First, the included studies were diverse, differing in therapeutic approach, delivery, and outcome measures. Second, all included studies were conducted in high-income countries. Therefore, these findings may not generalize to low- and middle-income countries. Third, we included articles only in English and peer-reviewed articles, which may have meant that relevant research was not included. Fourth, there were no tests of publication bias, which may be a factor biasing the findings. Finally, the inclusion criteria were broad, which could have led to the inclusion of heterogeneous outcomes. Despite these limitations, this study made a comprehensive assessment of different types of digital health interventions for suicide prevention among LGBTQ. This study identified the importance of digital health interventions and their feasibility and usability, which could be advantageous in designing and implementing policies for adequately functioning and strengthening suicide prevention intervention.

## Conclusion

In recent years, digital health interventions have emerged as innovative intervention delivery platforms for mitigating STBs and fostering increased help-seeking behavior in suicide prevention efforts. While there are existing digital health interventions tailored to vulnerable populations like LGBTQ, the development and implementation of such interventions should align with a heightened emphasis on empirically supported mHealth technology. Currently, research in this domain has predominantly focused on high-income countries; nevertheless, the rapid advancements in the digital landscape within LMICs necessitate the immediate development and deployment of such interventions in these regions.

## Figures and Tables

**Table 1:** PICO framework

**P**	Population	• LGBTQ
**I**	Intervention	• Digital health interventions for the prevention of suicide
**C**	Comparator	• No digital health interventions
**O**	Outcomes	• Suicidal ideation • Help-seeking intention of suicidal ideation

**Table 2:** List of keywords and full search strategy

Search query	Keywords (searched as titles, abstract, and keywords)
1	“LGBTIQA+” OR “LGBTQ” OR “LGBT” OR “lgbtq+” OR “sexual minority” OR “sexual minorities” OR “gender minority” OR “gender minorities” OR “lesbian” OR “gay” OR “bisexual” OR “transgender” OR “queer”
2	“Suicide” OR “suicidal” OR “suicidal attempt” OR “suicidal ideation” OR “suicidal plan” OR “suicidality”
3	“digital” OR “technology” OR “technologies” OR “internet” OR “internet-based” OR “web-based” OR “social media” OR “social network” OR “SNS” OR “social networking service” OR “telehealth” OR “ai” OR “artificial intelligence” OR “vr” OR “virtual reality” OR “eHealth” OR “telemedicine” OR “mHealth” OR “mobile phone”
4	“intervention” OR “treatment” OR “therapy” OR “implement” OR “application” OR “program” OR “strategy” OR “implementation”

**Table 3:** Characteristics of the studies

First author and year of publication (setting)	Aim	Age, Mean (SD)	Study Population	Study type	Sample size	Intervention type	Digital platform	Outcome
Han, M., et.al., 2023 (China)	This study aimed to assess the effectiveness of a digital multicomponent intervention in promoting help-seeking for mental health issues in LGBTQ+ young adults.	22.2 (2.8)	Bisexual, Gay/lesbian, Pansexual, Asexual, straight	RCT	137	Online animated psychoeducational videos, online facilitator-led group discussions, and electronic help-seeking brochures. 5 modules in the intervention video were all presented from the perspective of LGBTQ+ populations, and the characters used were LGBTQ+ friendly.	Video conference	Improvement in help-seeking intention of suicidal ideation. Participants in the intervention condition had significant improvement in the help-seeking intention of suicidal ideation at post-discussion and 3 months follow-up.
Kirchner, S., et. al., 2022 (Onsite Austria, Online Germany)	This randomized controlled trial (RCT) hypothesizes that participants would benefit from selected “Its Gets Better Project” (IGBP) videos in terms of a reduction in suicidal ideation (primary outcome), improvements in help-seeking intentions, identity challenges, and hopelessness.	18.9 (2.2)	Lesbian, Gay, Bisexual, Questioning, Queer, Pan, Asexual and romantic	RCT	483	Videos featuring a young cisgender woman and man to help prevent suicide and strengthen sexual identity.	Intervention was delivered through social media sites like LGBTQ Facebook groups, Reddit, Tumblr pages, Instagram.	There was no effect of the videos on the total intervention group regarding suicidal ideation. An indirect preventive effect on suicidal ideation at T2 through the degree of identification with the protagonist in the video was observed. There was improvement in help-seeking intentions in the intervention group (T2: MC = 0.25 [95% CI 0.15 to 0.35], p < 0.001; MD = 0.28 [95% CI 0.01 to 0.54], p < 0.05, d = 0.09).
Pachankis, JE., et.al., 2020, (USA)	To identify scalable interventions for improving sexual minority mental health and health-risk behavior, this study tested the efficacy of two self-guided online writing interventions – expressive writing and self-affirmation.	23.7 (3.1)	Bisexual, Lesbian, Gay, Pansexual, Asexual, Questioning, Other	RCT	108	Brief online writing interventions (One writes about a stressful or traumatic event for about 20 minutes per day for several days. Self-affirmation (Three brief scenarios reflecting common instances of severe sexual minority stress: family rejection, religious community rejection, and school-based victimization. Participants in the self-affirmation group were tasked with a daily 20-minute writing exercise for three consecutive days)	Email with instruction	Self-affirmation exerted improvement in suicidal ideation. For control, the SIDAS score in baseline was 12.90 and dropped to 10.43, whereas in intervention, after three months of follow-up, the SIDAS score dropped from 15.98 to 6.46.
Dubov, AA., et.al., 2021 (USA)	This study aims to beta test the usability of an evidence-informed mobile health (mHealth) suicide prevention phone app, Trans Life.	33.4 (9.5)	Transwomen, Transmen	Qualitative –Usability study	16	Trans life mobile app with features like several mood logging and cognitive behavioral therapy (CBT) m-health interventions.	Mobile application	This application is an engaging and acceptable mental health intervention. This provides initial support for the acceptability and usability of the application.
Biernesser, C., et.al., 2023 (USA)	This study aims to develop Flourish, a digital suicide prevention intervention for LGBTQ+ youth who have experienced online victimization.	15.7 (1.3)	Lesbian, Gay, Bisexual, Pansexual, Queer, other	Qualitative – Usability study	33	Flourish digital platform through co-design. Flourish on a spectrum ranging from no automation, i.e., a phone call with a counselor, to full automation, i.e., artificial intelligence or chatbot.	Mural, a digital whiteboard space.	This actively engaged LGBTQ+ youth and found this acceptable. All youth thought Flourish would be easy to use without a parent. All were interested (n=10) in using the app because its tone was positive, caring, empathetic, authentic/relatable, and helpful for coping skills. Similarly, all participants thought the app would be easy to learn, and a few (n=2) suggested including some instructions to use, significantly improving adolescents’ understanding. Many participants did not track their mood daily (4 days per week on average, SD 2.7).
